# Rescue of a spontaneous subcapsular hepatic hematoma associated with HELLP syndrome: A case report

**DOI:** 10.1097/MD.0000000000040285

**Published:** 2024-11-01

**Authors:** Chenze Yan, Zhong Jia, Yuanwei Liu, Weijiang Zhou, Heshan Zhou

**Affiliations:** a The Fourth School of Clinical Medicine, Zhejiang Chinese Medical University, Hangzhou First People’s Hospital, Hangzhou, China; b Department of Hepatobiliary Surgery, Affiliated Hangzhou First People’s Hospital, School of Medicine, Westlake University, Hangzhou, China; c Department of Reproductive Endocrinology, Hangzhou Women’s Hospital, Hangzhou, China; d Department of Radiology, Affiliated Hangzhou First People’s Hospital, School of Medicine, Westlake University, Hangzhou, China.

**Keywords:** HELLP syndrome, pregnancy-induced hypertension, spontaneous subcapsular hepatic hematoma

## Abstract

**Rationale:**

Spontaneous subcapsular hepatic hematoma (SSHH) is a rare yet severe complication of hemolysis, elevated liver enzymes, and low platelet count (HELLP) syndrome that can lead to life-threatening situations for both the mother and fetus. Determining an appropriate therapeutic strategy remains challenging, as it involves choosing between surgery, microinvasive percutaneous transhepatic drainage, or conservative treatment alone. Further successful cases are needed to support the optimal option. We retrospectively collected a patient’s clinical record and imaging data to elucidate the natural progression, response to noninvasive treatment, and outcome of SSHH associated with HELLP syndrome.

**Patient concerns:**

A 34-year-old woman, who underwent a cesarean section due to suspected fetal distress, developed SSHH accompanied by the potential risk of rupture and deteriorated serology within the first 24 hours after delivery.

**Diagnoses:**

Emergency blood routine examination, serum biochemistry analysis, and computed tomography of the abdomen revealed a SSHH associated with HELLP syndrome.

**Interventions:**

The main rescue measures included the use of corticosteroids, antihypertensive drugs, and platelet transfusion.

**Outcomes:**

A positive effect on the patient’s condition was exhibited within 24 to 48 hours. The majority of the subcapsular hepatic hematoma could be absorbed without any sequelae over a period of 7 months.

**Lessons:**

For patients with pregnancy-induced hypertension, there is a heightened risk of HELLP syndrome occurrence in subsequent pregnancies. The assessment and treatment of SSHH should be conducted by an experienced multidisciplinary team. In addition to timely delivery, the administration of corticosteroids, usage of antihypertensive medication, and platelet transfusion are necessary, particularly within the first 48 hours if the patient’s condition permits. This approach would provide valuable insights for further therapeutic decisions and facilitate a preliminary prognosis assessment.

## 1. Introduction

The academic term “HELLP” syndrome is an abbreviation for a syndrome consisting of hemolysis (H), elevated liver enzymes (EL), and low platelet count (LP) that occurs in mid to late pregnancy or the early postpartum period. This event was first observed in 1954 and was formally proposed by Dr Louis Weinstein in 1982.^[1,2]^ The syndrome is characterized by hepatic endothelial dysfunction, platelet aggregation and consumption, hepatocellular necrosis and death, or even the development of subcapsular hepatic hematoma or its rupture, ultimately contributing to both maternal and fetal mortality (24% and 60%, respectively).^[3,4]^ It is considered one of the various manifestations of preeclampsia because approximately 70% to 80% of cases with HELLP syndrome coexist with it.^[5]^ Herein, we describe a postpartum woman who suffered from a spontaneous subcapsular hepatic hematoma (SSHH) and experienced successful rescue, aiming to understand the natural outcome of SSHH under nonsurgical management, gain preliminary experience in rescue techniques, and provide tips for decision-making regarding therapeutic strategies through reviewing related literature.

## 2. Case history

**Figure 1.**
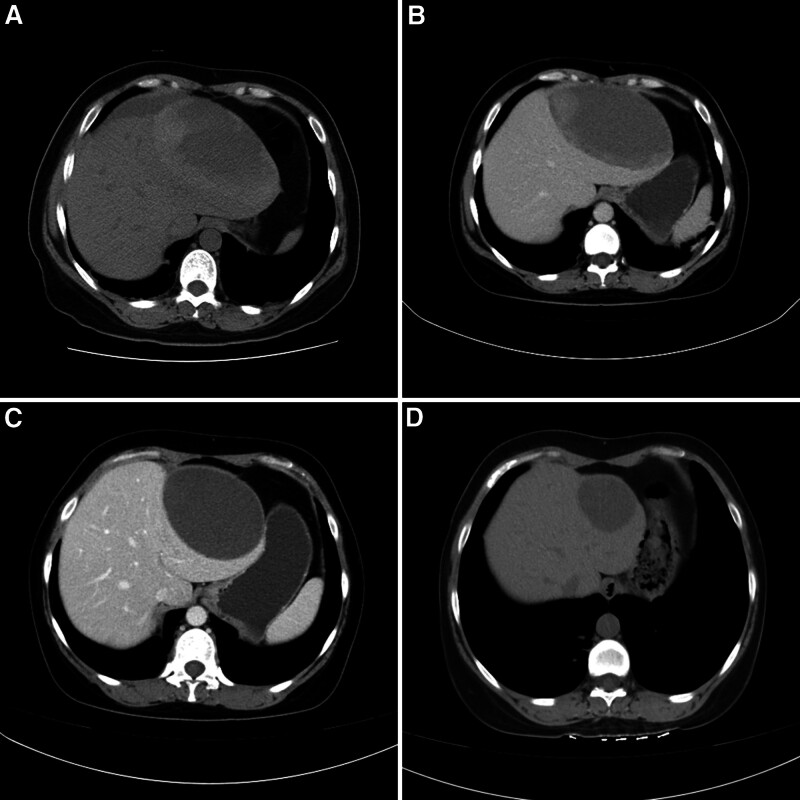
(A) CT scan revealed a subcapsular hepatic hematoma predominantly located in the left lobe, characterized by heterogeneous contents and minimal blood accumulation. (B) CT scan revealed resolution of the previously identified hepatic hematoma with retraction on the 8th day of hospitalization. (C) CT scan revealed significant reduction in size of the hepatic hematoma at a 2-wk follow-up. (D) CT scan revealed substantial absorption of the majority of the hepatic hematoma at a 7-mo follow-up. CT = computed tomography.

A 34-year-old woman with pregnancy-induced hypertension (PIH) for 4 years underwent an emergency lower-segment cesarean section under general anesthesia due to suspected fetal distress at 34 weeks of gestation. The procedure was successful, resulting in the delivery of a live baby boy. On the day following delivery, she suddenly experienced chest tightness at midnight. Apart from her history of PIH with blood pressure ranging from 146 to 160/90 to 110 mm Hg, there were no other relevant medical records available. Emergency blood routine examination and serum biochemistry analysis revealed decreased hemoglobin levels, LP, and ELs including alanine aminotransferase, aspartate aminotransferase, and lactate dehydrogenase (LDH) (Table 1). Emergency computed tomography of the abdomen revealed a large SSHH mainly located in the left lobe (Fig. 1A). Unfortunately, the serological parameters deteriorated rapidly within the first 6 hours after onset of symptoms leading to her immediate transfer to a higher-level general hospital from the local obstetrics and gynecology hospital where she initially presented. The deterioration of serological parameters and the increasing size of SSHH reached their peaks respectively within the first 24 hours after delivery (Tables 1 and 2). After a multidisciplinary discussion, the diagnosis of HELLP syndrome was made. Furthermore, a multidisciplinary rescue team was established to implement appropriate treatment measures, including the administration of corticosteroids, antihypertensive medications, transfusion of red blood cells and platelets, as well as plasma exchange. After an initial 24 to 48 hours of close dynamic surveillance, the serological parameters and the size of the SSHH were successfully reversed (Tables 1 and 2), leading to clinical stabilization with no major fluctuations. On the eighth day of hospitalization, just 2 days prior to discharge, an abdominal computed tomography reexamination revealed a decrease in the size of the SSHH (Fig. 1B). At a follow-up examination after discharge at 2 weeks post-hospitalization, there was a notable reduction in the size of the SSHH (Fig. 1C). At 7 months post-hospitalization follow-up visit, most of the SSHH had been absorbed with no sequelae (Fig. 1D). After 1 year of follow-up assessment period elapsed since her discharge from hospital care, she had fully recovered her health and vitality and successfully resumed her job position.

**Table 1:** The major serological parameters in different time.

	Hours to symptom onset											
	−12	−8	0	+3	+6	+11	+14	+20	+29	+34	+58	+84
Hemoglobin (g/L)	130	129	121	112	115	109	107	67	N/A	75	79	92
Platelet count (n × 10^9^/L)	137	111	96	81	88	96	90	14	N/A	84	124	214
ALT (U/L)	25	38	96	140	253	287	339	N/A	N/A	318	298	231
AST (U/L)	33	N/A	162	222	352	385	436	N/A	395	291	194	89
LDH (U/L)	223	N/A	646	728	765	637	631	N/A	381	314	293	313

“−” means how many hours before symptom onset; “+” means how many hours after symptom onset.

ALT = alanine aminotransferase, AST = aspartate aminotransferase, LDH = lactate dehydrogenase, N/A = not available.

**Table 2:** The size of the SSHH in different times.

	Hours to symptom onset			
	+14	+23	+40	+63
The size of the SSHH	16.2 × 7.3 × 10.8 cm	15.2 × 8.2 × 13.2 cm	12.8 × 9.8 × 7.3 cm	12.5 × 6.5 × 10.9 cm

“+” means how many hours after symptom onset.

SSHH = spontaneous subcapsular hepatic hematoma.

## 3. Discussion

The HELLP syndrome, characterized by hemolysis, impaired liver function, LP, and the rarity of SSHH or its rupture during pregnancy or after delivery, has gained increasing clinical importance over the years. The incidence rate of the HELLP syndrome in pregnant women ranges from 0.2% to 0.8%.^[6]^ However, its pathogenesis remains incompletely understood. It is widely accepted that the innate immune response to gestational status and gestational hypertension might trigger an excessive maternal inflammatory response and enhanced endothelial activation.^[7]^ High-risk factors associated with this condition include advanced maternal age (≥35 years), multiparity, family history of HELLP syndrome, and poorly controlled PIH.^[8]^ In this case, the patient presented multiple high-risk factors including being nearly 35 years old, having a subsequent pregnancy after previous delivery, coexisting PIH along with fluctuating blood pressure peaking at 160/110 mm Hg accompanied by chest tightness. Therefore, it is crucial to exercise heightened vigilance for high-risk pregnancies especially when combined with gestational hypertension or preeclampsia. Currently, with an enhanced comprehension and heightened awareness of integrated diagnosis for the HELLP syndrome, a precise diagnosis of the HELLP syndrome can be achieved if appropriate differential diagnosis has been conducted.^[9]^ According to Table 1, it was observed that the degree of elevation in alanine aminotransferase, aspartate aminotransferase, and LDH appeared to be more severe compared to the decline in hemoglobin concentration and platelet count during the initial symptoms associated with HELLP syndrome. This implies that alterations in liver enzyme levels serve as earlier and more sensitive indicators for detecting the presence of HELLP syndrome. However, diagnosing HELLP syndrome is not paramount here since patient outcomes rely on timely termination of pregnancy, real-time assessment of clinical condition, justified judgment regarding maternal changes, and implementation of suitable therapeutic strategies. To the best of our knowledge, limited attention has been given to observing the evolutionary process of HELLP syndrome in previous studies. In this regard, we propose a chronological division of HELLP syndrome into 3 distinct phases as follows: acute phase characterized by deteriorating serological changes and potential enlargement of SSHH. In this phase, many impending and fatal clinical situations may emerge, such as hypovolemic shock, peripheral circulatory failure, risk of bleeding in any part, liver and kidney failure, SSHH rupture, and maternal or fetal death. The duration of this phase typically ranges from 1 to 3 days, emphasizing its significance for timely intervention; relative plateau phase where effective rescue measures lead to a second stage marked by slight fluctuations but an overall stable maternal condition. This period can extend from 3 days up to 2 weeks; recovery or sequelae phase during which maternal health generally improves or residual complications necessitate further treatment. This phase may persist for several weeks to months or longer. Notably, early identification and prompt intervention during the acute phase significantly influence prognosis. With regards to treatment strategies based on our perspective, emphasis should be placed on 4 key aspects: timely termination of gestational status, management of PIH, addressing current clinical concerns while proactively preventing potential risks ahead and managing any residual sequelae effectively to enhance maternal quality of life. These considerations facilitate informed therapeutic decision-making.

The spontaneous formation of a hepatic hematoma during pregnancy or within 40 hours after delivery is frequently associated with the HELLP syndrome. As a critical condition, it should be distinguished from other diseases such as hematopathy, liver tumors, and traumatic hematomas due to differences in etiology, pathogenesis, treatment strategies, and surgical timing. The subcapsular hepatic hematoma poses a greater risk compared to the central hepatic hematoma because of its heightened susceptibility to eventual rupture. In the early stages, the condition often progresses rapidly, leading to increased tension of the liver capsule. This is typically manifested as abdominal pain radiating to the shoulder or back, chest tightness due to restricted transdiaphragmatic movement, or gastrointestinal symptoms resulting from compression caused by a significant hematoma volume. Any factors leading to an elevation in intra-abdominal pressure, such as the contraction of abdominal wall muscles resulting from sudden movements or changes in posture, or uterine contractions during vaginal delivery, should be minimized to prevent the initiation or progression of SSHH and its subsequent rupture. If hematoma rupture occurs during pregnancy, conservative treatment carries a significantly higher mortality rate compared to surgical intervention.^[10]^ Therefore, immediate surgical intervention involving hemostasis by ligation or hepatectomy should be performed concurrently with cesarean section completion. Nevertheless, is surgical hemostasis always warranted in cases of subcapsular hepatic hematoma following delivery? Upon reviewing the literature, we identified a case of HELLP syndrome in which an emergency liver transplantation was performed due to SSHH following delivery, resulting in significant maternal morbidity and substantial financial burden.^[11]^ Possibly, the patient may have alternative options available for rescue. Therefore, we conducted a meticulous analysis of our reported case utilizing the original serological and imaging data associated with HELLP syndrome. It was revealed that the critical condition exhibited significant fluctuations primarily within the initial 24 hours following symptom onset. Fortunately, prompt implementation of rescue measures such as corticosteroid administration, antihypertensive medication usage, platelet transfusion, and correction of hypoalbuminemia demonstrated favorable effects during the first 24 to 48 hours. Nonetheless, early delivery was deemed essential as both a fundamental treatment and a prerequisite for subsequent rescue interventions. Furthermore, imaging examinations indicated deposition of fibrin-like material forming distinct partitions within the hepatic hematoma, suggesting an important indicator for its elimination and retraction. Additionally, persistent abdominal pain along with blunt liver edge angle or balloon-shaped morphology of the lesion accompanied by minimal effusion on the surface of the liver capsule may serve as potential indicators for impending rupture of subcapsular hepatic hematoma.^[12]^ These insights from our reported case could also aid in predicting imminent rupture of the hematoma.

The use of corticosteroids and the timing of their administration continue to be subjects of intense debate. Several clinical trials have failed to provide conclusive evidence regarding the superior efficacy of corticosteroids compared to placebo or other treatments in terms of clinical outcomes.^[13]^ Nevertheless, the vast majority of people still advocate for the cautious use of corticosteroids when strictly adhering to appropriate indications. The merits associated with their usage are evident in the following aspects: facilitating fetal lung development and maturation in women with HELLP syndrome between 24 and 34 weeks gestation, provided that maternal condition allows; elevating platelet count in cases where it poses a risk of bleeding (<50 × 10^9^/L); enhancing maternal survival rates during impending or early-stage liver failure; suppressing excessive immune responses during the acute phase of HELLP syndrome. In such instances, intravenous dexamethasone administered within 24 to 72 hours may effectively stabilize SSHH. Excessive deliberation over weighing pros and cons can sometimes lead to catastrophic consequences. Platelet transfusion is indicated prior to or following delivery, or in the presence of bleeding from any site, particularly when platelet count falls below 40 × 10^9^/L. Fresh frozen plasma transfusion may be necessary in the presence of coagulopathy. Exchange transfusion should be considered in cases of progressive elevation of bilirubin, declining hemoglobin or platelets, or ongoing deterioration in maternal condition. Antithrombin and glutathione have the potential to correct hypercoagulability, stimulate prostacyclin production, regulate thrombin-induced vasoconstriction, and improve fetal status. They offer an advantage over heparin by not increasing bleeding risk. However, determining the optimal balance between bleeding risk and therapeutic benefit remains a contentious issue in clinical practice. Encouragement is given for exploratory studies investigating antithrombin as a countermeasure against HELLP syndrome or summarizing experiences from successful clinical cases. Microinvasive percutaneous transhepatic drainage for SSHH is generally discouraged during the acute phase of HELLP syndrome due to its lack of efficacy and associated risks such as hemorrhage and infection. If the hematoma persists beyond 1 year postdelivery without absorption or transforms into an abscess caused by infectious pathogens, drainage can be considered to address its sequelae.

The limited experience of authors and reported cases regarding conservative treatment for SSHH associated with the HELLP syndrome necessitates a comprehensive summary of successful nonsurgical management as follows: SSHH requires a multidisciplinary approach involving obstetricians, physicians in the intensive care unit, surgeons specializing in hepatobiliary surgery, doctors in the department of radiology intervention, and vascular surgeons; surgical hemostasis or watchful waiting alone is not recommended for postpartum SSHH. Close dynamic surveillance for 24 to 48 hours is essential to assess the effectiveness of rescue measures and determine whether conservative treatment or surgery should be pursued; serum LDH levels serve as an important indicator reflecting maternal condition quality, particularly during the acute phase of HELLP syndrome; timely delivery and corticosteroid use significantly impact maternal and fetal survival rates while improving clinical outcomes during the acute phase of HELLP syndrome; establishing guidelines for rescue procedures using fishbone diagrams and closely monitoring the real-time dynamics of serology and the hematoma size is necessary to promptly adjust rescue protocols based on their trends.

## 4. Conclusion

Spontaneous subcapsular hepatic hematoma following delivery is a severe manifestation associated with the HELLP syndrome. Implementing conservative management strategies may offer a viable approach to achieve favorable clinical outcomes without long-term complications. The cornerstone of acute phase interventions, such as prompt delivery, corticosteroid administration, antihypertensive medication usage, and platelet transfusion, could potentially be effective in mitigating the progression of the HELLP syndrome within 24 to 48 hours from symptom onset.

## Author contributions

**Writing – original draft:** Chenze Yan.

**Writing – review & editing:** Chenze Yan, Zhong Jia, Yuanwei Liu, Weijiang Zhou, Heshan Zhou.

**Conceptualization:** Zhong Jia.

**Data curation:** Yuanwei Liu, Weijiang Zhou, Heshan Zhou.

## References

[1] Reubinoff BE Schenker JG. HELLP syndrome—a syndrome of hemolysis, elevated liver enzymes and low platelet count—complicating preeclampsia-eclampsia. Int J Gynaecol Obstet. 1991;36:95–102. 1683323 10.1016/0020-7292(91)90762-t

[2] Dusse LM Alpoim PN Silva JT Rios DR Brandão AH Cabral AC. Revisiting HELLP syndrome. Clin Chim Acta. 2015;451(Pt B):117–20. 26525965 10.1016/j.cca.2015.10.024

[3] Pokharel SM Chattopadhyay SK Jaiswal R Shakya P. HELLP syndrome—a pregnancy disorder with poor prognosis. Nepal Med Coll J. 2008;10:260–3. 19558067

[4] Mihu D Costin N Mihu CM Seicean A Ciortea R. HELLP syndrome—a multisystemic disorder. J Gastrointestin Liver Dis. 2007;16:419–24. 18193124

[5] Petca A Miron BC Pacu I. HELLP syndrome-holistic insight into pathophysiology. Medicina (Kaunas). 2022;58:326. 35208649 10.3390/medicina58020326 PMC8875732

[6] Abildgaard U Heimdal K. Pathogenesis of the syndrome of hemolysis, elevated liver enzymes, and low platelet count (HELLP): a review. Eur J Obstet Gynecol Reprod Biol. 2013;166:117–23. 23107053 10.1016/j.ejogrb.2012.09.026

[7] Gardikioti A Venou TM Gavriilaki E. Molecular advances in preeclampsia and HELLP syndrome. Int J Mol Sci. 2022;23:3851. 35409211 10.3390/ijms23073851 PMC8999044

[8] Lisonkova S Razaz N Sabr Y. Maternal risk factors and adverse birth outcomes associated with HELLP syndrome: a population-based study. BJOG. 2020;127:1189–98. 32189413 10.1111/1471-0528.16225

[9] Faridi A Rath W. Differentialdiagnose des HELLP-syndroms [differential HELLP syndrome diagnosis]. Z Geburtshilfe Neonatol. 1996;200:88–95. 8963890

[10] Escobar Vidarte MF Montes D Pérez A Loaiza-Osorio S José Nieto Calvache A. Hepatic rupture associated with preeclampsia, report of three cases and literature review. J Matern Fetal Neonatal Med. 2019;32:2767–73. 29478361 10.1080/14767058.2018.1446209

[11] Mazzola A Magro B Perdigao F. Acute liver failure and HELLP syndrome: a clinical case and literature review. Clin Res Hepatol Gastroenterol. 2021;45:101498. 32828747 10.1016/j.clinre.2020.07.005

[12] Luo MY Jia Z Cai Y Wan YF. Bleeding from the liver capsule—when to perform surgery. Chin Med J (Engl). 2017;130:1008. 28397736 10.4103/0366-6999.204100 PMC5407031

[13] Haram K Svendsen E Abildgaard U. The HELLP syndrome: clinical issues and management. A review. BMC Pregnancy Childbirth. 2009;9:8. 19245695 10.1186/1471-2393-9-8 PMC2654858

